# Managing Weed–Crop Interactions Enhances Chickpea (*Cicer arietinum* L.) Chemical Components

**DOI:** 10.3390/plants12173073

**Published:** 2023-08-27

**Authors:** Imtiaz Khan, Muhammad Ishfaq Khan, Saima Hashim, Muhammad Fawad, Aftab Jamal, Mahmoud F. Seleiman, Haroon Khan, Bakhtiar Gul, Zahid Hussain, Muhammad Farhan Saeed, Aurelio Scavo

**Affiliations:** 1 Department of Weed Science and Botany, Faculty of Crop Protection Sciences, The University of Agriculture, Peshawar 25130, Pakistan; imtiazkhan@aup.edu.pk (I.K.); mishfaq@aup.edu.pk (M.I.K.); saimahashim@aup.edu.pk (S.H.); fawadagrarian@aup.edu.pk (M.F.); haroonkhan@aup.edu.pk (H.K.); bakhtiargul@aup.edu.pk (B.G.); zhussainws@aup.edu.pk (Z.H.); 2 Department of Soil and Environmental Sciences, Faculty of Crop Production Sciences, The University of Agriculture, Peshawar 25130, Pakistan; aftabses98@gmail.com; 3 Department of Plant Production, College of Food and Agriculture Sciences, King Saud University, P.O. Box 2460, Riyadh 11451, Saudi Arabia; mseleiman@ksu.edu.sa; 4 Department of Environmental Sciences, COMSATS University Islamabad, Vehari Campus, Vehari 61100, Pakistan; 5 Department of Agriculture, Food and Environment (Di3A), University of Catania, 95123 Catania, Italy

**Keywords:** legume grain, weed control, allelopathy, plant extracts, mulching, herbicides, chemical components

## Abstract

Chickpea (*Cicer arietinum* L.) is a major pulse crop worldwide, renowned for its nutritional richness and adaptability. Weeds are the main biotic factor deteriorating chickpea yield and nutritional quality, especially *Asphodelus tenuifolius* Cav. The present study concerns a two-year (2018–19 and 2019–20) field trial aiming at evaluating the effect of weed management on chickpea grain quality. Several weed management practices have been here implemented under a factorial randomized complete block design, including the application of four herbicides [bromoxynil (C_7_H_3_Br_2_NO) + MCPA (Methyl-chlorophenoxyacetic acid) (C_9_H_9_ClO_3_), fluroxypyr + MCPA, fenoxaprop-*p*-ethyl (C_18_H_16_ClNO_5_), pendimethalin (C_13_H_19_N_3_O_4_)], the extracts from two allelopathic weeds (*Sorghum halepense* and *Cyperus rotundus*), two mulches (wheat straw and eucalyptus leaves), a combination of *A. tenuifolius* extract and pendimethalin, and an untreated check (control). Chickpea grain quality was measured in terms of nitrogen, crude protein, crude fat, ash, and oil content. The herbicides pendimethalin (Stomp 330 EC (emulsifiable concentrate) in pre-emergence at a rate of 2.5 L ha^−1^) and fenoxaprop-*p*-ethyl (Puma Super 7.5 EW (emulsion in water) in post-emergence at a rate of 1.0 L ha^−1^), thanks to *A. tenuifolius* control, showed outstanding performance, providing the highest dietary quality of chickpea grain. The herbicides Stomp 330 EC, Buctril Super 40 EC, Starane-M 50 EC, and Puma Super 7.5 EW provided the highest levels of nitrogen. Outstanding increases in crude protein content were observed with all management strategies, particularly with Stomp 330 EC and Puma Super 7.5 EW (+18% on average). Ash content was highly elevated by Stomp 330 EC and Puma Super 7.5 EW, along with wheat straw mulching, reaching levels of 2.96% and 2.94%. Crude fat content experienced consistent elevations across all treatments, with the highest improvements achieved by Stomp 330 EC, Puma Super 7.5 EW, and wheat straw mulching applications. While 2018–19 displayed no significant oil content variations, 2019–20 revealed the highest oil content (5.97% and 5.96%) with herbicides Stomp 330 EC and Puma Super 7.5 EW, respectively, followed by eucalyptus leaves mulching (5.82%). The results here obtained are of key importance in the agricultural and food sector for the sustainable enhancement of chickpea grain’s nutritional quality without impacting the environment.

## 1. Introduction

*Cicer arietinum* L. (chickpea or gram) is an annual winter crop belonging to the Fabaceae family. It is globally recognized as a nutritious crop, especially in Pakistan [1]. Chickpea seeds boast a nutrient-rich profile encompassing proteins, amino acids, fibers, calcium, iron, and phosphorus, while maintaining a low fat content (4–10%). These starchy seeds offer dietary advantages, such as combating ailments like diabetes, obesity, and cardiovascular diseases [2,3]. Despite its global cultivation over 14.5 million ha, yielding 14.8 million tons, the average productivity at 1 t ha^−1^ remains significantly below its estimated potential of 6 t ha^−1^ under optimal growing conditions [3,4].

However, this promising crop faces considerable threats from weeds, which diminish crop yields due to their competitive nature and allelopathic effects [5,6]. Chickpea’s relatively slower growth and limited leaf development make it susceptible to weed competition, adversely impacting productivity [7]. Numerous research reports identify weed damage in chickpea fields due to uncontrolled weed growth. For example, Nath et al. [8] reported a 64% reduction in chickpea yield due to weed infestation, while in another study, weed damage to chickpea crops in Tabriz, Kermanshah, and West Azerbaijan was estimated at 48.3%, 57%, and 36%, respectively [9]. Recently, Mahajan et al. [10] indicated that infestations of *Avena ludoviciana* Durieu and *Argemone mexicana* L. at densities of 15 and 17 plants m^−2^ caused an 83% and 48% reduction in chickpea yield, respectively, compared to weed-free conditions. Yield losses in chickpea may vary from 24% to 63%, depending on weed infestation levels [11]. However, when weeds are not effectively managed during the critical growth period of chickpea plants (35–60 days after emergence), yield losses as high as 88% [10] can be experienced. Furthermore, these yield losses highly depend on weed species composition, the degree of weed–crop competition, the weed infestation period, type and intervals of water application, and climatic conditions [5,6,12]. Major weeds infesting chickpea crops include *Anagallis arvensis* L., *Asphodelus tenuifolius* L., *Carthamus oxyacantha* L., *Convolvulus arvensis* L., *Cynodon dactylon* (L.) Pers, *Cyperus rotundus* L., *Fumaria indica* (Hausskn.) Pugsley, *Lathyrus aphaca* L., and *Medicago polymorpha* L. [1,7,8].

Weed infestation adversely affects chickpea grain’s dietary composition, particularly through a reduced nitrogen and crude protein content, thus compromising overall seed and grain quality [13]. Weeds compete for resources, impede nutrient uptake, and disrupt soil microbial activity, collectively diminishing chickpea’s nutritional value [11,14]. Understanding the unique mechanisms by which weeds, particularly *Asphodelus tenuifolius* Cav., affect the dietary status of chickpea grain is of key importance to adopt efficient weed management strategies.

Still, because of environmental issues, herbicide use needs to be reduced as much as possible. There are other eco-friendly alternatives to herbicides for weed management, such as crop rotation, the use of weed-suppressive cultivars [15], mulching, allelopathic extracts [16], or the combination of herbicides with allelopathic extracts. These agronomic techniques are allelopathic tools commonly adopted in agroecosystems [17]. The allelopathic extracts of various weeds were used by Mustafa et al. [18] to control weeds in chickpea. Despite the growing interest in enhancing the nutritional composition of chickpea grains through weed management strategies, there is still a notable research gap in our understanding that needs to be filled. The use of herbicides has emerged as a predominant weed management technique employed by farmers, especially pre-emergence herbicides like pendimethalin, in chickpea cultivation [1,7]. Moreover, herbicides are an effective control strategy against *A. tenuifolius*, the most noxious chickpea weed, and other associated weeds.

In this study, it was hypothesized that the application of several weed management strategies including herbicides, aqueous extracts of certain allelopathic weeds, and mulches can lead to improvements in the nutritional composition of *C. arietinum* grains, specifically targeting the control of *A. tenuifolius*, the most prevalent and detrimental weed in the studied area. Thus, the aims of the present study were to evaluate the influence of various weed management practices on the nutritional composition of chickpea grains, and to identify the most effective treatment for *A. tenuifolius* management in chickpea. The findings of this study may contribute to the sustainable cultivation of chickpea while providing quality nutritious food.

## 2. Results

The two-way ANOVA highlighted a significant effect of ‘weed management practice’ for all the variables under study, whereas the ‘year’ was only significant for the nitrogen and crude protein content (Table 1). No significant interactions were observed for any variable.

### 2.1. Nitrogen (N) Content

Significant variations were observed in the N content of chickpea grain among the different weed management treatments, with a similar trend across the two years (Table 2). Indeed, in both years, the highest N content was recorded under the herbicides Stomp 330 EC, Buctril Super 40 EC, Starane-M 50 EC, and Puma Super 7.5 EW, while the lowest one was the combined application of *A. tenuifolius* extract + Stomp 330 EC. Pooling over the years, the use of herbicides determined the highest levels of N in chickpea grain, followed by the mulching with wheat extract (+49% compared to control) and the allelopathic extract of *Cyperus rotundus* (+39%). On the contrary, *Eucalyptus* leaves mulching and the combined application of *A. tenuifolius* extract + Stomp 330 EC caused a N content reduction of −14% and −19%, respectively, compared to control. Averaged over weed management treatments, 2019–20 detected a higher N content than 2018–19 (Table 2).

### 2.2. Crude Protein Content

Significant differences regarding crude protein data were recorded among the weed management treatments (Table 3). In both years, all weed management treatments determined a higher crude protein content than control, with the herbicide Stomp 330 EC causing the highest level. On the average of years, the herbicides Stomp 330 EC and Puma Super 7.5 EW induced a +18% of crude protein content, as compared with control. Significant increases were also induced by the combined application of *A. tenuifolius* extract + Stomp 330 EC (+15%), *Eucalyptus* leaves mulching (+16%), and wheat straw mulching (+12%), as well as by the *Sorghum halepense* extract (+10%). As observed for N content, 2019–20 detected a higher crude protein content than 2018–19 (Table 3).

### 2.3. Crude Ash Content

The weed management treatments under study had differentiated effects on the ash content of chickpea grain (Table 4), with very similar results across the two years. Pooling over the years, as observed for the crude protein content, the herbicides Stomp 330 EC and Puma Super 7.5 EW determined the highest ash content (2.96% and 2.94%, respectively), followed by the wheat straw mulching (2.90%). The *Eucalyptus* leaves mulching, the allelopathic extract of *S. halepense*, and the combined application of *A. tenuifolius* extract + Stomp 330 EC also induced significant improvements in the ash content with respect to the control (+17%, +10%, and +9%, respectively). The year effect was not significant.

### 2.4. Crude Fat Content

Data show that the crude fat content in chickpea grain was significantly influenced by the weed management treatments and not by the year (Table 5). As observed for the crude protein content, all weed management treatments determined a higher fat content than control in both years, with the herbicides Stomp 330 EC and Puma Super 7.5 EW and the wheat straw mulching providing the highest levels (+22%, +22%, and +21%, respectively). Even in this case, the *Eucalyptus* leaves mulching, the combined application of *A. tenuifolius* extract + Stomp 330 EC, and the allelopathic extract of *S. halepense* also caused marked improvements of the fat content.

### 2.5. Oil Content

In 2018–19, no significant variations in the oil content of chickpea grain were observed across the weed management treatments (Table 6). On the contrary, in 2019–20, the herbicides Stomp 330 EC and Puma Super 7.5 EW exhibited the highest oil content (5.97 and 5.96%, respectively) in chickpea grain, followed by the *Eucalyptus* leaves mulching (5.82%). Pooling over the years, all weed management treatments under study increased the oil content, although without significant differences from each other’s. Also, the mean values did not differ between years.

## 3. Discussion

In light of these findings, it is revealed that the dietary quality of chickpea grain is substantially influenced by herbicides, mulching, and allelopathic extract. The use of herbicides Stomp 330 EC and Puma Super 7.5 EW, in particular, increased the crude protein, crude ash, fat, and oil contents of chickpea grain. Here, we discuss the influence of various treatments on dietary constituents of chickpea grain.

Nitrogen, a fundamental constituent of plants, plays a pivotal role in diverse physiological processes governing plant growth [19]. It is a major component of amino acids, the building of proteins, which helps in the synthesis of enzymes and other vital compounds [20]. In this study, the application of the herbicide Stomp 330 EC, Buctril Super 40 EC, Starane-M 50 EC, and Puma Super 7.5 EW consistently improved the N content of chickpea grain, while the combined application of *A. tenuifolius* extract with Stomp 330 EC led to the lowest N content. These outcomes imply that herbicides might effectively alleviate the N competition exerted by weeds, thereby favoring an enhanced nutrient assimilation by chickpea plants [21]. The substantial increase in N content associated with herbicide application reaffirms the potential of weed management practices to foster nutrient availability and optimize nutrient uptake, corroborating the existing literature [22]. Furthermore, wheat straw mulching and the allelopathic extract of *Cyperus rotundus* emerged here as noteworthy contributors to increase the N content. Their effects could be attributed to the gradual release of nutrients from wheat mulch in the former case, and to the improved growth, nodulation, and nitrogen fixation in the latter one [23]. Nonetheless, the lower N content in the control plots indicates the deleterious effect of weed competition on plant nutrient availability and uptake. Studies report that weeds absorb N from the soil, ultimately reducing the amount of available N for plants [24]. Additionally, on the one side, weeds can affect the symbiotic relationship between chickpea plants and nitrogen-fixing bacteria, while on the other side, they can also reduce the amount of N during natural fixation [23]. However, it should be considered that the N content in chickpea grain is dependent on the specific efficacy of weed control practice; hence, there is likely to be a directly proportional relationship between N content and weed control [25].

Chickpea seeds are known to contain a significant amount of crude protein. In this research, the application of the herbicides Stomp 330 EC and Puma Super 7.5 EW has led to a noteworthy increase in chickpea’s crude protein content. This might be attributed to the herbicides’ ability to alleviate weed competition and enable cultivated plants to allocate more resources towards protein synthesis [26]. Similarly, the role of herbicides in enhancing protein formation by reducing nutrient competition between crops and weeds has been documented by Dong et al. [27]. Furthermore, here we demonstrated that other sustainable treatments (i.e., the combined application of *A. tenuifolius* extract with Stomp 330 EC, *Eucalyptus* leaves and wheat straw mulching, as well as *Sorghum halepense* extract) showed an outstanding contribution to enhancing the crude protein content of chickpea grain. This effect could be explained by the reduction in weed competition for nutrients through allelopathic plant extracts and mulching, thereby enabling chickpea plants to allocate more resources towards protein synthesis [28]. Similarly, Merga et al. [21] reported a positive impact of weed control on crude protein content in chickpea grains, with crude protein levels reaching approximately 34% of the dry matter. Therefore, the observed higher crude protein content in this study is likely due to the absence of weed competition among crops.

The observed higher crude ash contents in chickpea seeds following the application of Stomp 330 EC and Puma Super 7.5 EW may be attributed to their effective weed-suppressive ability. By effectively managing weed populations, it is likely that these herbicides create a weed-free environment where chickpea plants face less competition for vital resources such as nutrients, water, and space [21]. Consequently, they are better poised to allocate resources towards their own growth and nutrient accumulation, potentially resulting in the observed increase in ash content. In contrast, in the control plots, the intense weed–crop competition may exert considerable stress on chickpea plants, leading to compromised nutrient uptake and allocation. This phenomenon corroborates with the findings of Hoover et al. [29], who establish a clear correlation between weed competitiveness and the mineral composition of chickpea grains. In addition to the ash content, the presence of weeds indirectly influences other nutritional components such as protein, fat, and oil contents [30].

For instance, our findings on the crude fat content, another important nutritional aspect of chickpea grain, are consistent with the study of Khan et al. [1], who also found that severe weed competition significantly deteriorates the nutritional quality of chickpea seeds. A further recent study reveals that intense weed infestation could have a negative effect on the dietary quality of chickpea grains by reducing the soil nutrients and interfering with nutrient uptake [31]. Moreover, Khan et al. [32] found a differentiated crude fat content in green gram (*Vigna radiata* (L.) R. Wilczek) seeds due to the application of the herbicides atrazine, isoproturon, metribuzin, and sulfosulfuron. Chickpea seeds have the highest oil content among all the pulses, ranging from 3% to 10% of the total dry seed weight [33]. However, weeds such as wild onion are known to considerably decrease its levels in chickpea grains. Here we found that weed control practices, especially herbicides, played a key role in improving oil content of chickpea seeds. A similar finding is also reported by Wood and Grusak [33].

In addition to the weed–crop interactions, weather conditions could have affected numerous aspects of chickpea grain quality. Indeed, different plant species show a differentiated response to stress factors and adaptation to different climatic conditions [34]. Weather data show that the average air temperatures during the 2018–19 growing season were 1 °C higher than 2019–20, and total rainfall was 112 mm higher in 2019–20 than in 2018–19. Our results, particularly the N and protein content, show significant variations across the years, whereas ash, fat, and oil content were similar in both growing seasons. It is reasonable to assume that the variations in N content could be due to some extent to the differences in weather conditions [35]. Interestingly, the year × weed management strategy interaction was not significant on the dietary constituents of chickpea, indicating that the performances of the proposed weed control techniques were not affected by weather across the years.

## 4. Materials and Methods

### 4.1. Plant Material

Chickpea (*Cicer arietinum* L.) cv. “Chattan”, an Asian high-yielding genotype, was used as a test crop in this study. Healthy and mature seeds with 95% germination capacity were obtained from the National Agricultural Research Centre (NARC) of Islamabad, Pakistan. This cultivar is recommended for both rainfed and irrigated field conditions.

### 4.2. Study Area

**Figure 1:**
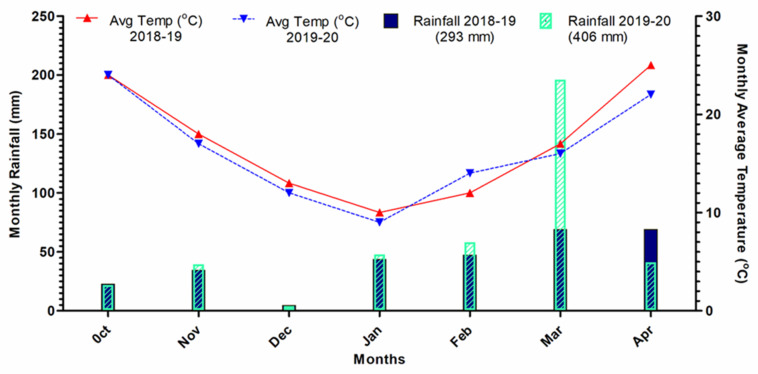
Weather data (monthly average temperatures (°C) and rainfall (mm)) at the experimental site during the two growing seasons of chickpea crop.

Field trials were carried out for two consecutive years (2018–19 and 2019–20) at the Agriculture Research Station of Karak, Khyber Pakhtunkhwa (Pakistan), located between 33°11′45″ N and 77°09′74″ E at an altitude of 950 m above sea level. This region is devoted to the cultivation of a variety of field crops, including cereals (maize, pearl millet, sorghum, wheat, and barley), ground nuts, chickpea, and mustard, typically grown under rainfed conditions. The area has a semi-arid climate with an average annual rainfall of 450 mm, and mean air temperatures ranging from 45 °C in the summer to 10 °C in the winter season [36]. Soil samples from the 0–20 cm layer were collected in each plot before the start of the experiment using a soil auger. The samples were air dried, crushed to pass through a 2 mm sieve, mixed to make a composite sample, labeled, and stored in a plastic bag. Standard laboratory methods were used for physical and chemical characterization [37]. The soil type, a Calcaric Luvisols according to the World Reference Base (WRB) system of soil taxonomy, had a sandy-loam texture (60% sand; 25% silt; 15% clay), alkaline reaction (pH 8.2), was calcareous (164.3 g kg^−1^ lime), and non-saline (0.3 dS m^−1^ electrical conductivity). It showed a low organic matter (8.6 g kg^−1^) and nutrient content (total N 0.1 g kg^−1^; AB-DTPA assimilable phosphorous 4.3 mg kg^−1^ and potassium 129 mg kg^−1^). Weather data show that the average air temperature during the growing season 2018–19 was 1 °C higher than 2019–20, whereas total rainfall in 2019–20 was 112 mm higher than in 2018–19, exhibiting a considerable variation in the weather pattern across the years (Figure 1).

### 4.3. Experimental Setup and Crop Management

Nine weed management treatments were compared to an untreated control for their effects on the nutritional composition of chickpea grain: four herbicides, two allelopathic plant extracts, two mulches, and a combined herbicide + allelopathic extract application. In both years, the experiment was arranged in a randomized complete block design (RCBD) with 3 replications. The plot size was 3 × 1.5 m (4.5 m^2^) and the total number of plots was 30 (10 treatments × 3 replications), for a total net experimental area of 135 m^2^ (4.5 m^2^ plot size × 30 plots). Plots with herbicide application were separated from each other by a 2 m border.

Field was ploughed twice and levelled to ensure good drainage, prevent waterlogging, and promote proper root development. Chickpea was sown at a rate of 50–60 kg seed ha^−1^, corresponding to 28 seeds m^−2^, at 5–8 cm soil depth in a 30 cm and 15 cm row-to-row and plant-to-plant distance, respectively. Sowing and crop harvest were respectively carried out on 13 October 2018 and 16 April 2019 during the first growing season, and on 18 October 2019 and 26 April 2020 during the second growing season. During both years, no fertilizers were used. Insecticide Lambda-cyhalothrin Ec 250mL (2.5%) was applied for cutworm (*Agrotis ipsilon* (Noctuidae: Lepidoptera)) control. However, no disease symptoms were observed during both years.

#### 4.3.1. Herbicide Application

Herbicide Puma Super 7.5 EW (fenoxaprop-*p*-ethyl) was used in post-emergence at a rate of 1.0 L ha^−1^, Stomp 330 EC (pendimethalin) in pre-emergence at a rate of 2.5 L ha^−1^, Buctril Super 40 EC (bromoxynil + MCPA) in post-emergence at the rate of 1.4 L ha^−1^, and Starane-M 50 EC (fluroxypyr + MCPA) at a rate 1.02 L ha^−1^. Herbicides were applied with a hand-operated knapsack sprayer with 12 L capacity. The dosages were those recommended by producers.

#### 4.3.2. Preparation and Application of Allelopathic Plant Extracts

Fresh biomass of *Sorghum halepense* (L.) Pers. and *Cyperus rotundus* L., two well-known allelopathic weeds, was randomly collected in nearby fields of the experimental location from plants at full maturity stage. The biomass was shade-dried up to constant weight and powdered with a mechanical grinder. A dry biomass of 125 g of each species was added to 1 L distilled water and soaked for 24 h at 25 °C. Then, the suspension was filtered through a muslin cloth and the final aqueous extract was applied in post emergence, 3–4 weeks after chickpea sowing, at an application rate of 20 L ha^−1^. This concentration has been found to be effective in inhibiting the growth of other plants and can be used to evaluate the allelopathic potential of the extract [38].

#### 4.3.3. Mulch Application

Mulch can prevent the germination and growth of weed seeds both serving as a physical barrier (and thus by blocking light and limiting the moisture) and chemically by producing compounds called allelochemicals that prevent weed growth [39]. Eucalyptus (*Eucalyptus camaldulensis* Dehnh) leaves and wheat (*Triticum aestivum* L.) straws were used as allelopathic dead mulches. Apparently, allelochemicals produced by *Eucalyptus* leaves can inhibit the growth of many crops and weeds [40]. Furthermore, the allelopathic chemicals contained in wheat straws could retard weed growth [39].They were placed between chickpea rows, making a two-inch-deep layer mulch on the soil surface in each plot, respectively.

#### 4.3.4. Herbicide and *A. tenuifolius* Aqueous Extract Tank Mixing

The aqueous extract solution of *A. tenuifolius* was prepared at a ratio of 4:60, i.e., 4 g powdered sample was added in 60 mL distilled water, and kept for 24 h at room temperature. After complete soaking, the extract was filtered to remove any solid particles to obtain a clear solution. Half dose of Stomp 330 EC was mixed with the allelopathic extract of *A. tenuifolius*. The herbicide and extract solution were stirred to ensure proper mixing and applied in post-emergence after 30 days of crop sowing on the targeted weeds within the crop rows by using a hand-operated knapsack sprayer with 12 L capacity.

### 4.4. Chemical Analyses of Chickpea Grain

The grain samples of chickpea were collected at harvest stage and thoroughly washed with distilled water, dried at room temperature for 24 h, then oven dried at 65 °C till constant weight. The nitrogen and crude protein contents were determined by modified Kjeldhal method [41]. The samples were ground to a homogenous powder by using a grinding machine and were digested in H_2_SO_4_ and di-acid (HNO_3_:HClO_4_ at 9:4 ratio) by following the standard procedure of Chapman and Pratt [42]. The crude protein content was calculated by multiplying the amount of nitrogen with appropriate factor (6.25).

$$\mathrm{Crude}\;\mathrm{protein}\;\mathrm{content}\;(\%)=\mathrm{nitrogen}\;\mathrm{percentage}\times 6.25\;\left(\mathrm{factor}\;\mathrm{for}\;\mathrm{chickpea}\right)$$

Fat was extracted from ground samples according to AOAC method 920.39 [43] using anhydrous ether in a Soxhlet apparatus (Extraction system B-811, BÜCHI Labortechnik AG., Flawil, Switzerland). Finally, the percentage was computed using the following formula:

$$\%\;\mathrm{Crude}\;\mathrm{fat}\;\mathrm{content}=\frac{\mathrm{Beaker}\;\mathrm{wight}\;+\;\mathrm{Ether}\;\mathrm{extract}\;\mathrm{weight}\;-\;\mathrm{Beaker}\;\mathrm{weight}}{\mathrm{Sample}\;\mathrm{weight}}\times 100$$

Chickpea crude ash content was determined by the AOAC method 942.05 [43]. Samples were weighed (2 g) in separate, pre-weighed porcelain crucibles, and placed in a preheated furnace (600 °C) for 2 h. Crucibles were then transferred to a desiccator, cooled, and reweighed. Sample weight remaining after ignition of a 2 g sample was regarded as ash content. The percentage of crude ash was then determined using the following formula:

$$\%\;\mathrm{Ash}\;\mathrm{content}=\frac{\mathrm{Weight}\;\mathrm{of}\;\mathrm{ash}\;\left(W_{3}\;-{\;W}_{1}\right)}{\mathrm{Sample}\;\mathrm{weight}}\times 100$$

where W_1_ = initial weight of the crucible, and W_3_ = final weight of the crucible with ash.

To determine the oil content, chickpea seeds were selected randomly one by one from all the treatments. The oil content was extracted from the obtained chickpea seeds using an oilseed extracting machine at the PCSIR (Pakistan Council for Scientific and Industrial Research) labs in Peshawar, and the oil was weighed. The detailed descriptions of the above-mentioned analytical methods can be found in Raza et al. [41].

### 4.5. Statistical Analysis

The collected data were statistically analyzed by analysis of variance (ANOVA) as appropriate for RCBD, using the statistical package Statistix ver. 8.1 (Statistix8.1, Tallahassee, FL, USA). In detail, a two-way ANOVA model was carried out to test the significance of ‘weed management practice’, ‘year’ and their interaction. The homogeneity of variance and normality were respectively tested with the Bartlett’s test and by graphically inspecting the residuals. Percentage values were subjected to arcsine square root transformation (Bliss) to meet the ANOVA basic assumptions. If the *F*-values were significant, the means were compared with the Fisher’s protected LSD test at α = 0.05.

## 5. Conclusions

In light of the results, it is evident that severe infestation of *A. tenuifolius* substantially reduces the dietary constituents of chickpea grain in the studied area, as observed in the untreated control plots. Interestingly, adequate nutrients or irrigation water supplies to crop are not expected to obtain nutritional-quality chickpeas unless weeds are not properly managed. Among the weed management strategies proposed under rainfed conditions, the use of post-emergence herbicides had outstanding performance in supressing weed growth and improving the nutritional quality of chickpea grain (nitrogen, crude protein, crude fat, oil, and ash). However, keeping in view the health hazards and environmental concern of herbicides, the reduced dose of pendimethalin in combination with the aqueous extract of *A. tenuifolius* or the adoption of the other eco-friendly weed management practices under an integrated strategy are suggested to sustainably increase the dietary quality characteristics of chickpea grains. These practices are economically feasible and generally acceptable by the area’s farming community. In future steps, we aim to focus on the specific relationships between nutritional quality parameters and chickpea grain yield, as well as on the weed control data and species composition related to the proposed weed management strategy.

## Figures and Tables

**Table 1:** *F*-Fisher values of main factors and their interactions resulting from two-way analysis of variance (ANOVA) on nutritional quality of chickpea grain.

	Source of Variation		
	Weed Management Practice (WP)	Year (Y)	(WP) × (Y)
df	9	1	9
Nitrogen content	61.06 ***	8.04 **	0.02 ns
Crude protein content	8.32 ***	7.43 **	0.59 ns
Crude ash content	24.08 ***	0.08 ns	0.18 ns
Crude fat content	20.13 ***	0.03 ns	0.02 ns
Oil content	3.33 **	1.91 ns	0.77 ns

*F*-Fisher values are referred to Bliss-transformed data; df: degrees of freedom; *** and ** indicate statistical significance at *p* ≤ 0.001 and *p* ≤ 0.01, respectively; ns: not significant.

**Table 2:** Effect of different weed management treatments on nitrogen content (%) in chickpea grain.

Treatment	2018–19	2019–20	Mean
Stomp 330 EC (Pre-emergence)	8.00 ± 0.14 a	8.44 ± 0.07 a	8.22 ± 0.10 a
Buctril Super 40 EC	8.04 ± 0.27 a	8.48 ± 0.07 a	8.26 ± 0.17 a
Puma Super 7.5 EW	7.73 ± 0.35 a	8.17 ± 0.45 ab	7.95 ± 0.40 a
Starane-M 50 EC	8.01 ± 0.22 a	8.43 ± 0.31 a	8.22 ± 0.27 a
Mulching with wheat straw	6.50 ± 0.41 b	6.94 ± 0.49 bc	6.72 ±0.45 b
Mulching with *Eucalyptus* leaves	3.63 ± 0.25 de	4.07 ± 0.51 de	3.85 ± 0.38 d
Allelopathic extract of *Cyperus rotundus*	6.03 ± 0.38 b	6.47 ± 0.27 c	6.25 ± 0.33 b
Allelopathic extract of *Sorghum halepense*	4.56 ± 0.30 c	5.00 ± 0.29 d	4.78 ± 0.30 c
Extract of *Asphodelus tenuifolius* + Stomp 330 EC	3.44 ± 0.45 e	3.88 ± 0.37 e	3.66 ± 0.41 d
Untreated check (Control treatment)	4.29 ± 0.14 cd	4.73 ± 0.36 de	4.51 ± 0.25 c
Mean	6.02 ± 0.29 b	6.46 ± 0.37 a	

Different letters within a column indicate significant differences at *p* ≤ 0.05 (LSD test).

**Table 3:** Effect of different weed management treatments on crude protein content (%) in chickpea grain.

Treatments	2018–19	2019–20	Mean
Stomp 330 EC (Pre-emergence)	17.92 ± 0.44 a	18.08 ± 0.53 a	18.00 ± 0.48 a
Buctril Super 40 EC	16.48 ± 0.34 d	16.72 ± 0.37 abc	16.60 ± 0.36 d
Puma Super 7.5 EW	17.82 ± 0.54 ab	18.03 ± 0.31 a	17.92 ± 0.43 a
Starane-M 50 EC	15.96 ± 0.29 de	16.70 ± 0.51 abc	16.33 ± 0.40 de
Mulching with wheat straw	16.85 ± 0.43 cd	17.43 ± 0.44 abc	17.14 ± 0.44 bc
Mulching with *Eucalyptus* leaves	17.36 ± 0.53 abc	17.86 ± 0.54 a	17.61 ± 0.54 ab
Allelopathic extract of *Cyperus rotundus*	15.28 ± 0.47 ef	16.13 ± 0.61 bc	15.70 ± 0.54 ef
Allelopathic extract of *Sorghum halepense*	16.70 ± 0.31 cd	16.95 ± 0.39 abc	16.82 ± 0 35 cd
Extract of *Asphodelus tenuifolius* + Stomp 330 EC	17.32 ± 0.37 abc	17.63 ± 0.49 ab	17.47 ± 0.43 ab
Untreated check (Control treatment)	14.36 ± 0.29 f	16.10 ± 0.54 c	15.23 ± 0.42 f
Mean	16.61 ± 0.40 b	17.16 ± 0.47 a	

Different letters within a column indicate significant differences at *p* ≤ 0.05 (LSD test).

**Table 4:** Effect of different weed management treatments on crude ash content (%) in chickpea grain.

Treatment	2018–19	2019–20	Mean
Stomp 330 EC (Pre-emergence)	2.94 ± 0.21 a	2.97 ± 0.13 a	2.96 ± 0.17 a
Buctril Super 40 EC	2.60 ± 0.15 c	2.63 ± 0.09 cd	2.62 ± 0.11 c
Puma Super 7.5 EW	2.93 ± 0.23 a	2.94 ± 0.11 a	2.94 ± 0.17 a
Starane-M 50 EC	2.44 ± 0.11 d	2.48 ± 0.10 de	2.46 ± 0.11 d
Mulching with wheat straw	2.91 ± 0.18 ab	2.89 ± 0.15 ab	2.90 ± 0.17 ab
Mulching with *Eucalyptus* leaves	2.81 ± 0.18 b	2.81 ± 0.12 abc	2.81 ± 0.15 b
Allelopathic extract of *Cyperus rotundus*	2.47 ± 0.10 d	2.50 ± 0.14 de	2.49 ± 0.12 d
Allelopathic extract of *Sorghum halepense*	2.64 ± 0.13 c	2.64 ± 0.16 cd	2.64 ± 0.15 c
Extract of *Asphodelus tenuifolius* + Stomp 330 EC	2.63 ± 0.21 c	2.67 ± 0.10 bcd	2.65 ± 0.16 c
Untreated check (Control treatment)	2.43 ± 0.14 d	2.36 ± 0.13 e	2.40 ± 0.14 d
Mean	2.68 ± 0.16 a	2.69 ± 0.12 a	

Different letters within a column indicate significant differences at *p* ≤ 0.05 (LSD test).

**Table 5:** Effect of different weed management treatments on crude fat content (%) in chickpea grain.

Treatment	2018–19	2019–20	Mean
Stomp 330 EC (Pre-emergence)	4.91 ± 0.28 a	4.88 ± 0.24 a	4.90 ± 0.26 a
Buctril Super 40 EC	4.32 ± 0.31 e	4.28 ± 0.22 cde	4.30 ± 0.27 de
Puma Super 7.5 EW	4.87 ± 0.28 a	4.87 ± 0.24 a	4.87 ± 0.26 a
Starane-M 50 EC	4.24 ± 0.03 e	4.20 ± 0.19 de	4.22 ± 0.11 e
Mulching with wheat straw	4.83 ± 0.02 a	4.84 ± 0.21 ab	4.84 ± 0.11 a
Mulching with *Eucalyptus* leaves	4.69 ± 0.04 b	4.73 ± 0.17 ab	4.71 ± 0.11 ab
Allelopathic extract of *Cyperus rotundus*	4.47 ± 0.21 d	4.47 ± 0.22 cd	4.47 ± 0.22 cd
Allelopathic extract of *Sorghum halepense*	4.59 ± 0.01 c	4.59 ± 0.26 bc	4.59 ± 0.14 bc
Extract of *Asphodelus tenuifolius* + Stomp 330 EC	4.71 ± 0.27 b	4.71 ± 0.20 ab	4.71 ± 0.24 ab
Untreated check (Control treatment)	4.00 ± 0.22 f	4.00 ± 0.23 e	4.00 ± 0.23 f
Mean	4.56 ± 0.17 a	4.56 ± 0.22 a	

Different letters within a column indicate significant differences at *p* ≤ 0.05 (LSD test).

**Table 6:** Effect of different weed management treatments on the oil content (%) in chickpea grain.

Treatment	2018–19	2019–20	Mean
Stomp 330 EC (Pre-emergence)	5.70 ± 0.12 a	5.97 ± 0.31 a	5.84 ± 0.22 a
Buctril Super 40 EC	5.45 ± 0.30 a	5.34 ± 0.23 de	5.39 ± 0.27 a
Puma Super 7.5 EW	5.68 ± 0.23 a	5.96 ± 0.24 a	5.82 ± 0.24 a
Starane-M 50 EC	5.39 ± 0.30 a	5.26 ± 0.28 e	5.33 ± 0.29 a
Mulching with wheat straw	5.59 ± 0.14 a	5.68 ± 0.22 bc	5.64 ± 0.18 a
Mulching with *Eucalyptus* leaves	5.60 ± 0.05 a	5.82 ± 0.26 ab	5.71 ± 0.16 a
Allelopathic extract of *Cyperus rotundus*	5.56 ± 0.19 a	5.50 ± 0.28 cd	5.33 ± 0.24 a
Allelopathic extract of *Sorghum halepense*	5.47 ± 0.21 a	5.61 ± 0.28 bc	5.54 ± 0.24 a
Extract of *Asphodelus tenuifolius* + Stomp 330 EC	5.32 ± 0.16 a	5.61 ± 0.19 bc	5.47 ± 0.17 a
Untreated check (Control treatment)	3.53 ± 1.32 b	4.75 ± 0.24 f	4.14 ± 0.78 b
Mean	5.53 ± 0.30 a	5.55 ± 0.25 a	

Different letters within a column indicate significant differences at *p* ≤ 0.05 (LSD test).

## Data Availability

The data presented in this study are available on request from the first corresponding author.
